# Variation in resting strategies across trophic levels and habitats in mammals

**DOI:** 10.1002/ece3.8073

**Published:** 2021-10-06

**Authors:** Ishana Shukla, A. Marm Kilpatrick, Roxanne S. Beltran

**Affiliations:** ^1^ Ecology and Evolutionary Biology University of California Santa Cruz CA USA

**Keywords:** activity patterns, ethogram, human impacts, predator–prey, rest, species interactions

## Abstract

Mammals must carefully balance rest with other behaviors that influence fitness (e.g., foraging, finding a mate) while minimizing predation risk. However, factors influencing resting strategies and the degree to which resting strategies are driven by the activities of predators and/or prey remain largely unknown. Our goal was to examine how mammalian resting strategies varied with trophic level, body mass, and habitat. We reviewed findings from 127 publications and classified the resting strategies of terrestrial and aquatic mammalian species into three categories: social (e.g., resting in groups), temporal (e.g., resting during the day), or spatial (e.g., resting in burrows). Temporal strategies were most common (54% of cases), but the prevalence of strategies varied with body mass and among trophic levels. Specifically, lower trophic levels and smaller species such as rodents and lagomorphs used more spatial and social resting strategies, whereas top predators and larger species used mostly temporal resting strategies. Resting strategies also varied among habitat types (e.g., rainforest vs. grassland), but this was primarily because closely related species shared both habitats and resting strategies. Human presence also affected resting strategies at all trophic levels but most strongly influenced top predators through shifts in rest timing. Human‐induced behavioral changes in rest patterns cascade to modify behaviors across multiple trophic levels. These findings advance our fundamental understanding of natural history and ecology in wild animals and provide a roadmap for future comparative studies.

## Graphical abstract

Despite the potentially large fitness consequences of resting in a suboptimal time or place, behavioral ecology research tends to focus on the active portions of species' daily patterns. We found that the prevalence of resting strategies varied with body mass and among trophic levels (e.g., lower trophic levels and smaller species such as rodents used more spatial and social resting strategies, whereas top predators and larger species used mostly temporal resting strategies). We also found that human presence affected resting strategies at all trophic levels but most strongly influenced top predators through shifts in rest timing.

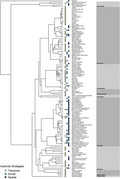

## 1 INTRODUCTION

Rest, a dormant state associated with reduced responsiveness (i.e., sleeping, quiet inactivity), is a vital part of mammalian life (Siegel, 2008). However, various levels of reduced sensory responsiveness during rest and inactivity may leave prey vulnerable to predation (Lima et al., 2005) and reduce opportunities for foraging and acquiring a mate (Brown, 1988). Thus, individuals must carefully optimize the timing, location, and duration of rest where marginal fitness gains are highest and costs are lowest (Brown, 1988). Despite the potentially large fitness consequences of resting in a suboptimal time or place, behavioral ecology research tends to focus on the active portions of species' daily patterns. Additionally, factors that modify resting patterns are commonly studied in laboratory settings rather than in the wild, where ecological drivers such as trophic level and habitat play a fundamental role (Acerbi et al., 2008; Rattenborg et al., 2017; Voirin et al., 2014). As a result, the degree to which resting behaviors of mammals are driven by top‐down (predator controls prey resting strategies) or bottom‐up (prey determines predator resting strategies) factors is not well understood (Pace et al., 1999).

The risk allocation hypothesis suggests that animals select behaviors based on the potential for endangerment (Lima & Bednekoff, 1999), which influences species‐specific resting strategies. Specifically, to reduce predation risk, animals can change locations to rest, change the timing of rest, rest in groups, or employ a combination of these strategies. Each strategy carries costs and has differential impacts on predation risks, especially with varying levels of vigilance (Lima et al., 2005). For example, some desert rodents that rest in the relative safety of burrows are more vulnerable to predation when actively foraging, whereas northern elephant seals *Mirounga angustirostris* are thought to be more vulnerable when they are inactive due to lack of protective resting habitat in the open ocean (Mitani et al., 2010). Additionally, predator–prey interactions can result in coupled activity patterns as prey attempt to limit predator exposure while predators attempt to maximize access to prey (Brown et al., 1999; Hunter & Skinner, 1998; Li et al., 2005). However, if prey can rest in refuges, then, somewhat paradoxically, prey can safely rest during peak predator activity periods, and shift foraging and other activities to times when predators are less active. Thus, strategy use may differ among species due to intrinsic traits (e.g., body mass, specific genetic controls like chronotype) or due to extrinsic factors like habitat or species interactions (Siegel, 2009), or a combination of factors. The frequency of these resting strategies has yet to be synthesized for wild mammals.

**FIGURE 1:**
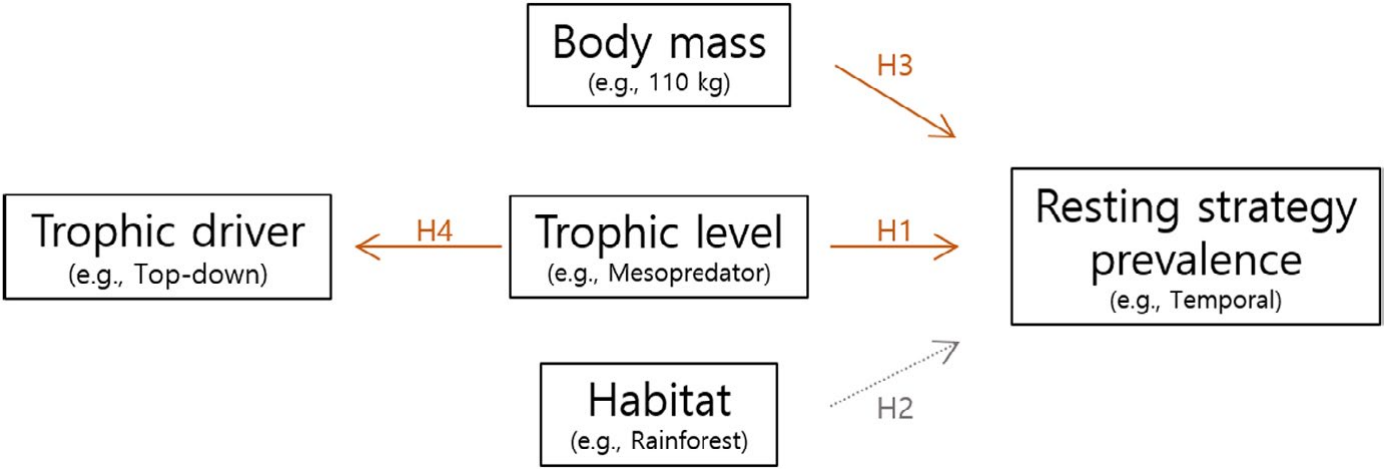
The suite of hypotheses tested to understand drivers of resting strategy prevalence. Hypotheses listed in orange were supported by the data whereas hypotheses listed in gray were not

Our objectives were to examine factors influencing mammal resting strategies and how strategies were influenced by humans. We tested a suite of four hypotheses to determine how resting strategy varied by habitat, trophic level, body mass, and the interactions between these factors (Figure 1). First, we hypothesized that resting strategies would vary among trophic levels because of different selective pressures including prey availability, predation risks, and human threats. For instance, lower trophic levels such as herbivores have comparatively higher rates of predation, yet much of their food is constant across space and time (Figure 1: H1). Second, we hypothesized resting strategies would differ among habitat because habitat structure determines the potential benefits of spatial and temporal strategies (Mazel et al., 2015) (Figure 1: H2). Specifically, we hypothesized that areas with limited protection from predators, such as aquatic habitats, savannas, grasslands, or deserts, would be associated more frequently with social and temporal resting strategies. On the other hand, we anticipated that tropical and temperate forests may be associated with less or different resting strategies because the abundance and variety of 3‐dimensional vegetation stratification in forests offer protection from both aerial and terrestrial predators (Ellison et al., 2019). Third, we hypothesized that larger species would be more likely to utilize temporal strategies than spatial strategies because many spatial refugia from predators (e.g., burrows) are more difficult for large animals to use as they are energetically costly (Capellini et al., 2008) (Figure 1: H3).

In addition to examining factors influencing the frequency of temporal, spatial, or social resting strategies, we examined whether resting strategies were influenced by higher trophic levels, lower trophic levels, both, the same trophic levels, or humans (hereafter: “trophic drivers”), and how this varied among trophic levels. We hypothesized that while humans would influence the resting strategies of all trophic levels, their effect would be largest on top predators (Figure 1: H4). While apex predators have no predation risks per se, human presence, urbanization, and other consumptive activities pose a disproportionally large threat to predators (Gaynor et al., 2018; Hill et al., 2020). We hypothesized that this landscape of fear would result in an increased proportion of temporal resting strategies in apex predators.

## 2 MATERIALS AND METHODS

We searched for publications on sleep and other forms of rest through Web of Science, Google Scholar, and JSTOR using the search terms: “mammal sleep predation,” “sleep predator avoidance,” “human disturbance nocturnal,” “human disturbance diurnal,” “mammal inactivity pattern,” “mammal resting pattern,” “mammal resting strategy,” or “mammal inactivity strategy.” We located additional publications by searching references cited and citing literature of each publication. When the publication's title or abstract included one or more of our search terms, we scanned the full paper to determine whether it met our eligibility requirements. To meet our criteria, the publication had to explicitly mention sleep, rest, and/or inactivity (i.e., studies focused on changes in the timing of foraging activity in relation to predators were not included, but studies focused on changes in the timing of resting activity were). In addition, the publication had to specifically reference an interaction between at least two species in which resting behavior was altered or shifted. This initial search result yielded 283 publications, and of those, 127 studies were deemed eligible and were included (*N* = 156 excluded). Most papers described patterns of sleep or resting behaviors when animals were less vigilant to predators. From each paper, we extracted information about resting strategies, habitat, and trophic level. Lastly, we extracted information on the trophic driver (bottom‐up, top‐down, both, self‐regulating, or human controlled) from each publication, which was determined by which species' resting behavior shifted more dramatically in the presence/absence of another.

**FIGURE 2:**
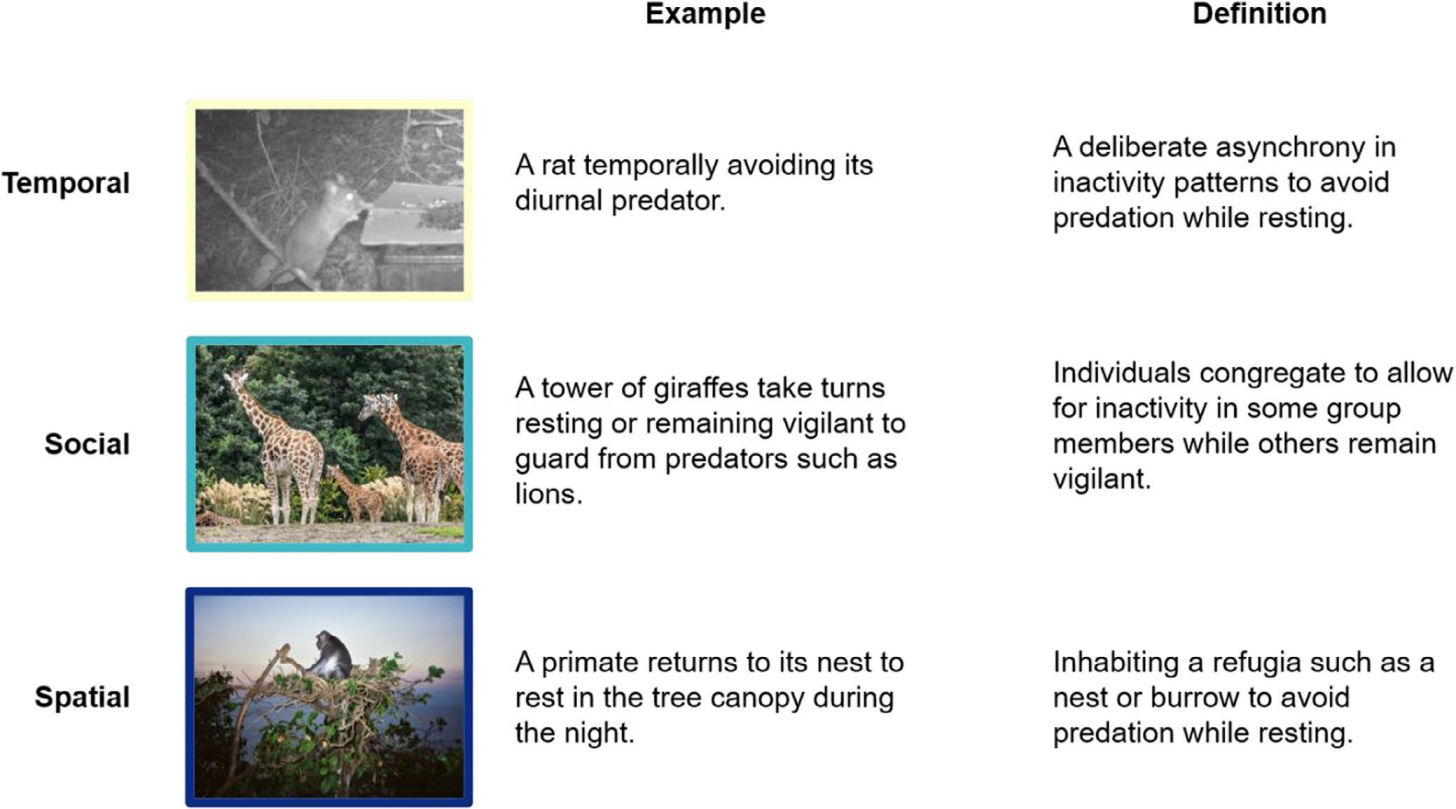
Examples and definitions for each of three resting patterns determined in our review: temporal, social, and spatial. Photo credits: Giraffes by William Murphy; Primates by Mo Riza

Here, we define resting strategies as specialized, repeated resting behaviors that facilitate predation avoidance while in a dormant state. For example, a resting strategy includes short‐term shifts in temporal inactivity timing, but not the evolutionary shift toward diurnality in mammals (Walls, 1942). We classified resting strategy variations into three categories: temporal, spatial, and social (Figure 2). Temporal strategies referred to a shift in the timing of rest. For example, Norway rats *Rattus norvegicus*, a typically nocturnal organism, shifted to a more diurnal pattern under the risk of red fox *Vulpes vulpes* predation (Arias‐Del Razo et al., 2011; Fenn & Macdonald, 1995). Spatial strategies included moving to a specific location where predation risk was lower for the purposes of resting, such as a burrow or nest. For instance, buffy‐headed marmosets *Callithrix flaviceps* chose resting sites with specific antipredatory features such as large crown cover and wider trunk diameter (Ferrari & Ferrari, 1990). We focused on behaviors within home ranges rather than at larger spatial scales. Finally, animals were classified as using a social resting strategy if they rested in groups of interspecific or intraspecific individuals, and benefited from increased vigilance and protection (Creel et al., 2014; Favreau et al., 2010; Fitzgibbon, 1990; Ritter & Bednekoff, 1994). For example, Angolan giraffes *Giraffa camelopardalis angolensis*, meerkats *Suricata suricatta*, and yellow mongooses *Cynictis penicillata* rested in groups and either took turns being vigilant or benefitted from an overall increased group vigilance (Burger et al., 2020; Roux et al., 2009). We created a categorical (binary‐coded) response variable for each of the three resting strategy variations to focus on the presence or absence of each strategy within each species. Each species was given a score (1 = present, 0 = absent) for each strategy, and thus, a single species could use more than one strategy.

We recorded the habitat and trophic level driver of each species in each publication. Habitats were classified as: aquatic, desert, grassland, tropical rainforest, savanna, temperate forest, and urban. For simplicity, grasslands encompass both chaparral and grassland habitat. Forest refers to temperate and coniferous forests, whereas rainforest refers to tropical forest. We distinguished between the two forest types because rainforests have higher levels of species diversity and richness (Gillman et al., 2015) and thus more interactions. Because recent urbanization has increased wildlife–human interactions (Shochat et al., 2006), we opted to include an “urban” habitat category where humans and wildlife occupy the same or adjacent areas. We also determined whether the resting strategies in each publication were driven by forces that were top‐down (driven by the trophic level above), bottom‐up (driven by the trophic level below), both (driven by an interplay of both top‐down and bottom‐up drivers), self‐regulating (driven by competition between species of the same trophic level), or human (driven by anthropogenic activity). These are referred to as “trophic drivers” throughout the manuscript. Species with multiple habitats or trophic levels were included in both categories.

We obtained the trophic level and body mass data for each species from the Smithsonian's Encyclopedia of Life (Parr et al., 2014). Specifically, we classified the trophic level of each species as: herbivore, omnivore, mesopredator, or top predator. Mesopredators were considered species with primarily carnivorous diets that are consumed by larger carnivores. Note that we list two species, coyotes and ocelots, in two separate trophic levels due to ecosystem‐level nuances (e.g., coyotes are generally mesopredators but can adopt the role of top predator when their natural predators are extirpated). We also extracted body mass data (geographic average across sexes, measured in kilograms) from the global database PanTHERIA (Jones et al., 2009). For the 19 species that were not included in the global database, we extracted body mass data from the Encyclopedia of Life.

We accounted for the nonindependence of species trait values (due to phylogenetic relatedness) in our analysis using a phylogenetic variance–covariance matrix (Blomberg et al., 2003; Hadfield & Nakagawa, 2010). We obtained a time‐scaled phylogenetic tree for mammals using vertlife.org full phylogenetic mammal trees (Upham et al., 2019) and trimmed the resulting trees to the subset of species in our review. We obtained 10 randomly sampled trees from the mammals birth–death node‐dated completed trees. We then computed phylogenetic multilevel Bayesian mixed models using the brm() function within the *brms* package in *R* to test whether resting strategies (the use of social, spatial, and temporal strategies per case) varied by habitat and/or trophic level while controlling for phylogenetic nonindependence and repeated measures for each species (Bürkner, 2017). Specifically, we used a categorical (multinomial) family with a logit link, normally distributed vaguely informative priors (*μ* = 0, *σ* = 20) for the coefficients, and 500 burn‐in iterations and 1,000 total iterations per chain for four chains, and used Rhat to assess convergence and mixing. We compared models with the expected log predictive density (elpd) values from the function *loo()* and present Δelpd values as the difference between the best fitting model and other models. Model selection results were identical using leave‐one‐out cross‐validation information criterion (loo_ic). Finally, we used the brm() function to test whether (log) mass varied by phylogeny and whether trophic driver varied by trophic level in our sample using the settings specified above.

## 3 RESULTS

**FIGURE 3:**
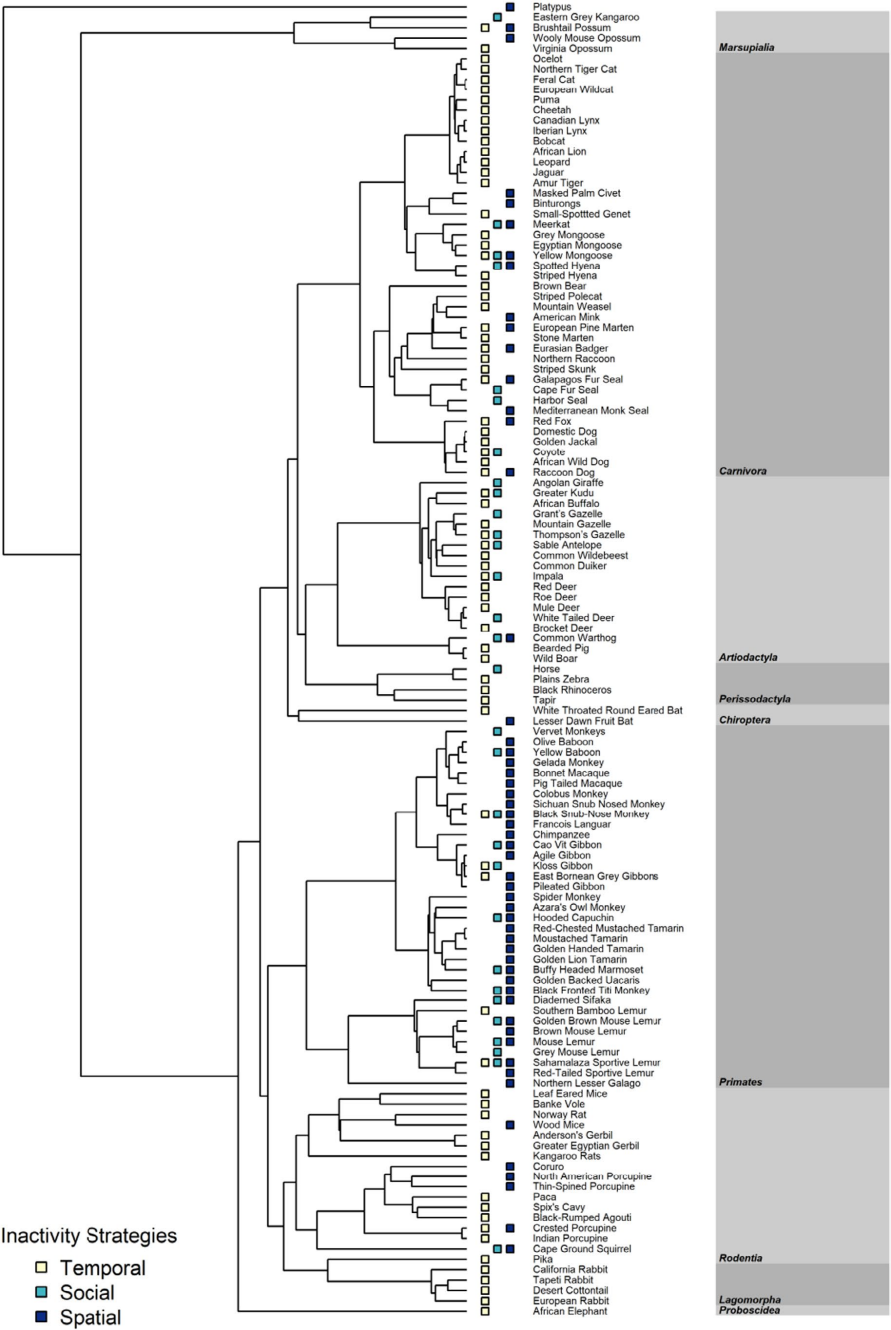
Phylogeny illustrating the relative frequency of three main resting strategies in 127 mammal species across nine orders (in gray rectangles)

Our final review included 127 papers published from 1980 to 2020 and contained data for 127 species across nine mammalian orders (Figure 3). The orders most represented were *Carnivora* (*N* = 44 species), *Primates* (*N* = 38), *Artiodactyla* (*N* = 19), and *Rodentia* (*N* = 18). Temporal strategies were the most common resting strategy (54% of cases), followed by spatial strategies (30%) and social strategies (16%). Most of the species studied primarily used one resting strategy (*N* = 105). Of the species that used multiple strategies, 27 species used two strategies and 3 species used three strategies. Within these species, the most common strategies were spatial and social, occurring in 25 and 20 species, respectively.

The best fitting models, across all phylogenetic trees, included either body mass or trophic level, with the model including mass having higher support (Table 1). In contrast, models with habitat, or combinations of multiple predictors, had weaker support than the null model (Table 1).

**TABLE 1:** Model comparison of mass, trophic level, and habitat as predictors of the prevalence of resting strategies in mammals

Explanatory variables			ΔlooIC
Mass	Trophic level	Habitat	
✓			0
	✓		1.38
			1.55*
	✓	✓	18.7
		✓	19.0
✓		✓	20.1
✓	✓	✓	20.7
✓	✓		21.1

Note

Values are presented as ΔlooIC (leave‐one‐out information criterion), the difference between the best fitting model (ΔlooIC = 0) and other models. The results from fitting models to ten phylogenetic trees are shown. Asterisk denotes the null model.

### 3.1 Body mass

**FIGURE 4:**
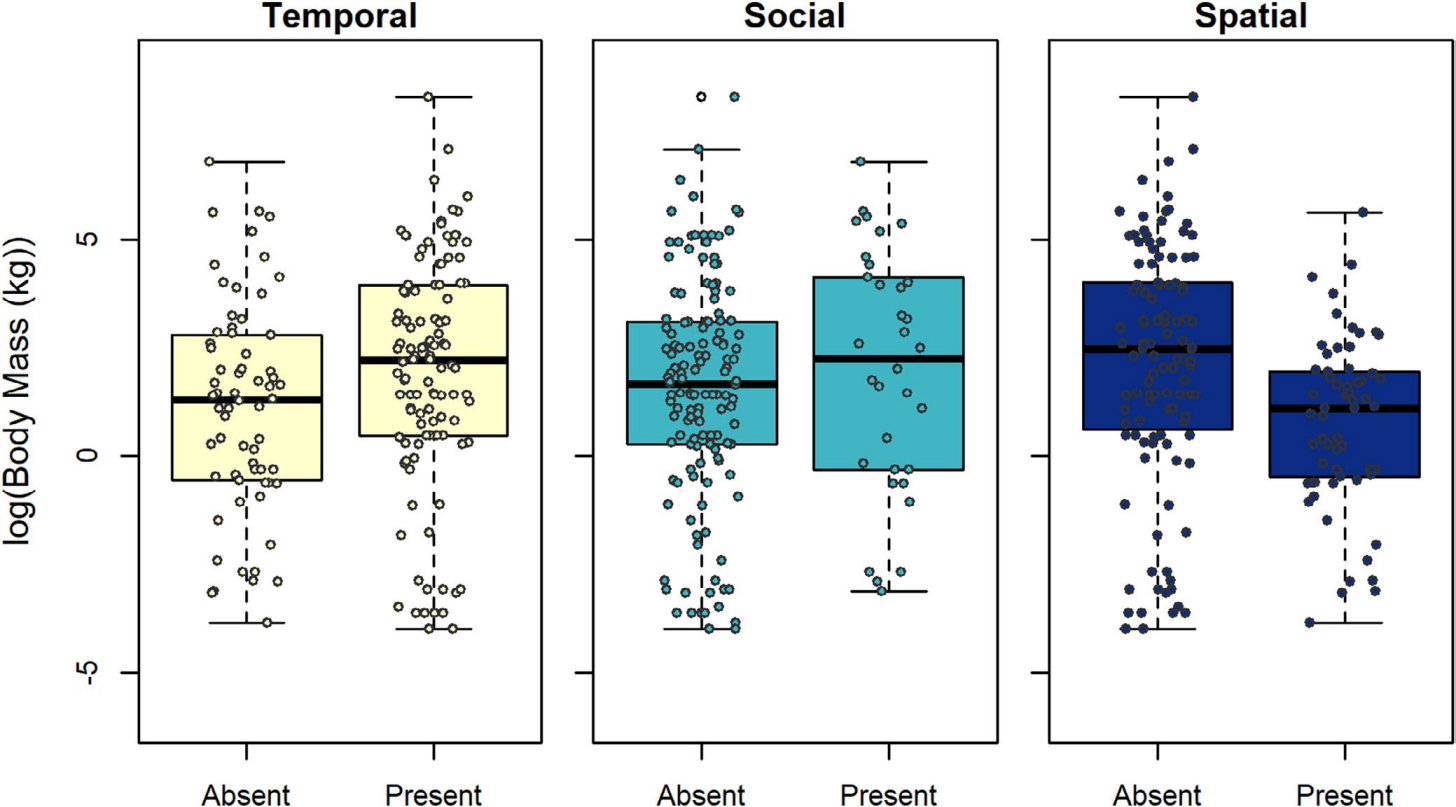
Body mass plotted against the presence or absence of temporal, social, and spatial strategies for rest in mammals (*N* = 127 species in each panel)

**FIGURE 5:**
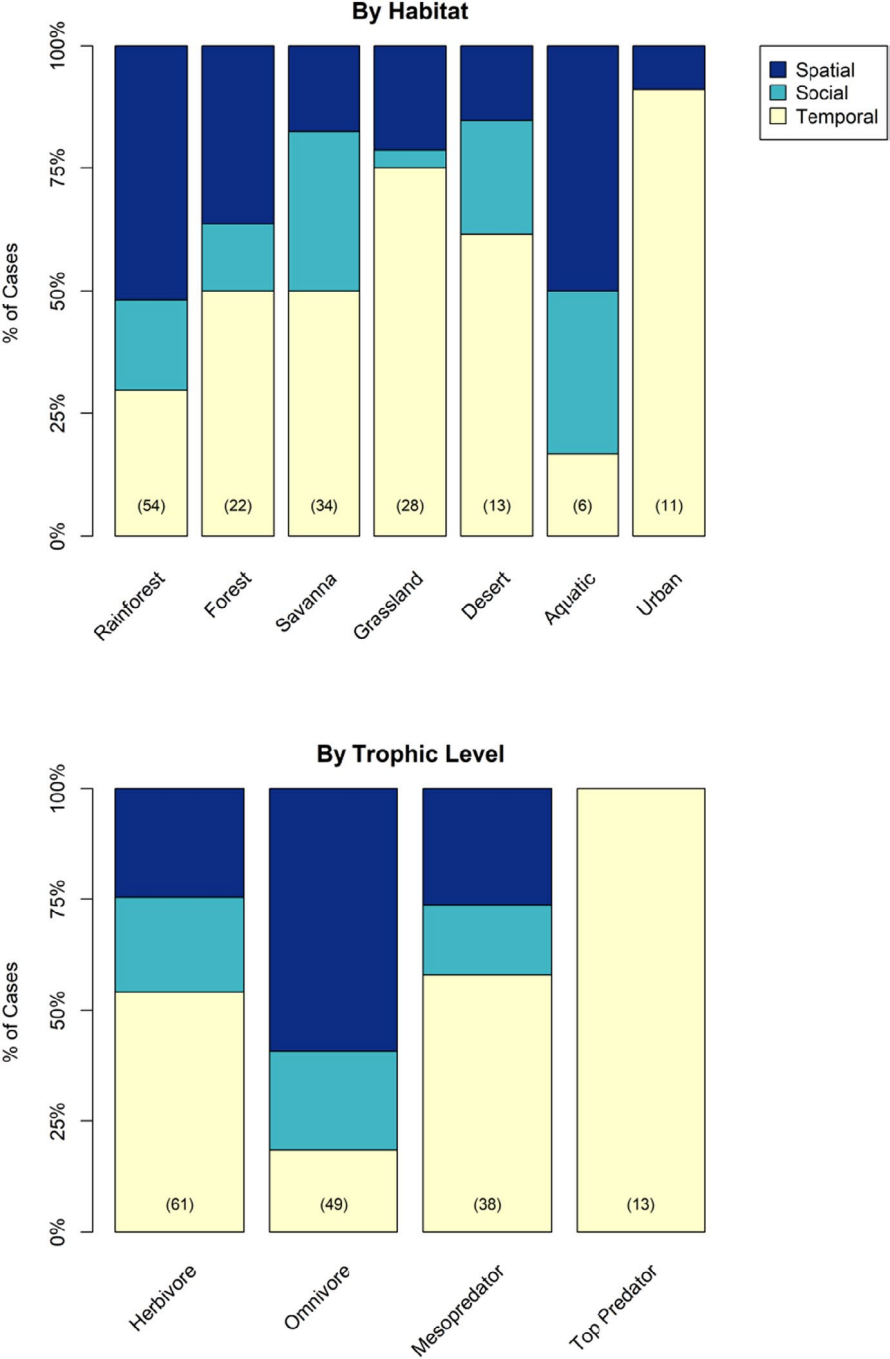
Mammalian resting behaviors categorized by study habitat and trophic level for the 127 species in Figure 3. Sample sizes in parentheses are 145 unique combinations of species and habitat (top panel) and 136 unique combinations of species and trophic level (bottom panel). Habitat categories are organized from left to right by the most to least cover available

Larger animals used spatial resting strategies less often than social strategies (Figure 4; Table S1). This pattern was due, in part, to top predators, which were larger than other species (Figure S1), not using spatial resting strategies (Figure 4). For example, larger animals, including predators such as lions, jaguars, as well as herbivores like tapirs and rhinoceros, used temporal strategies (Figure 3), whereas smaller mammals like wood mice *Apodemus sylvaticus* and ground squirrels *Xerus inauris* used spatial strategies by resting in refugia, such as burrows (Figures 3 and 5). Smaller animals were more able to use spatial resting strategies even in high‐risk, low‐cover habitats such as deserts (Edwards & Waterman, 2011).

### 3.2 Trophic level and habitat

There was partial support for resting strategies differing by trophic level (Table 1), with the dominant pattern being the use of temporal resting strategies by all predator species (Figure 5, Table S2). Although the majority of omnivorous species used spatial resting strategies (Figure 5), there was little support for a difference in resting strategies in this trophic level, because the group was primarily composed of a single taxon, primates (Figure 3).

Habitat was not supported as a predictor of resting strategies, alone or in combination with other predictors (Table 1; Table S3). Although more species in more complex habitats (rainforests and temperate forests) used spatial resting strategies than those in less complex habitats such as deserts and grasslands (Figure 5), much of the variation in resting strategy across habitats was due to phylogenetic correlations among species that had similar resting strategies in similar habitats (Figure 3). For example, rodents and lagomorphs frequently utilized temporal resting strategies and nearly all primate species used spatial resting strategies, whereas social resting strategies were common in artiodactyls (Figure 3).

### 3.3 Trophic drivers

**FIGURE 6:**
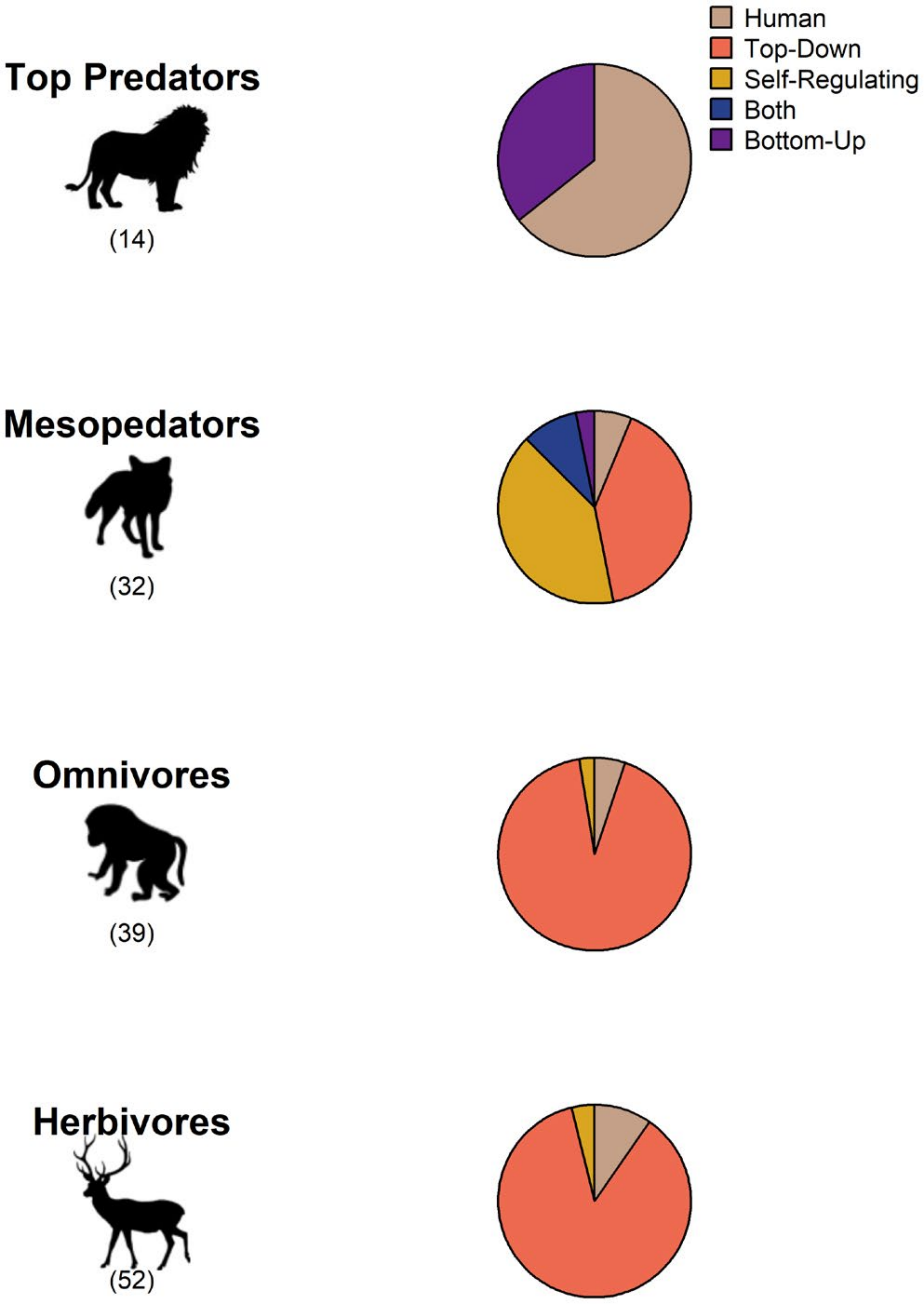
Factors influencing resting strategies in each trophic level. “Top‐down” indicates that resting patterns are determined by the trophic level above; “Self‐regulating” indicates that resting patterns are determined by competition between species of the same trophic level; “Bottom‐up” indicates that resting patterns are determined by the trophic level below; “Both” indicates that the resting pattern of a trophic level is influenced by an interplay of both top‐down and bottom‐up drivers; and “Human” indicates that resting patterns are driven by anthropogenic activity. Data represent 136 unique combinations of species and trophic level (*N* given in parentheses), from 127 species

The trophic driver of resting strategies differed among trophic levels (ΔlooIC = 54.3), with humans and bottom‐up forces (i.e., prey) playing a dominant role for top predators (Figure 6), while omnivorous and herbivorous mammals were influenced primarily by top‐down (i.e., predator) effects other than humans (Figure 6). Mesopredator resting behaviors were influenced primarily by top‐down and self‐regulating factors and to a lesser extent bottom‐up factors (Figure 6).

## 4 DISCUSSION

Activity patterns have been studied extensively, but studies often overlook rest, despite its clear ties to health and survival. Potential fitness consequences of rest include lost opportunities to forage, attract a mate, and, unless individuals rest in a spatial refuge, an increase in predation risk due to reduced vigilance. We found that mass and trophic level influence resting strategies among wild mammals. Although temporal avoidance was the most common resting strategy, spatial and social strategies were more common for smaller species and lower trophic levels. We also found that the drivers of resting strategies differed sharply among trophic levels, underscoring the interactions between resting strategies, predation risk, and foraging activity patterns.

### 4.1 Human impacts

We found that human presence affected mammalian resting strategies at all trophic levels but most strongly influenced top predators through shifts in rest timing. Humans contribute to a “landscape of fear,” which interferes with natural resting patterns and foraging strategies in many wild animals (Ciuti et al., 2012; Coppes et al., 2017; Suraci et al., 2019). Indeed, humans have artificially selected for particular chronotypes by harvesting animals, limiting food availability, and introducing light pollution (Helm et al., 2013; Martorell‐Barcelo et al., 2018). This directly alters antipredator behavior and risk allocation behavior, as human disturbance can shift temporal and spatial risk gradients from periods of short‐term risk to areas with long‐term high risk (Dröge et al., 2017). For example, commonly hunted ungulates such as elk *Cervus canadensis* change their rest timing to avoid human presence, especially during hunting seasons (Di Bitetti et al., 2008; Visscher et al., 2017). In species that face human hunting pressures year‐round, disrupted resting patterns become entrenched as new “normal” behaviors, despite their added energetic or nutritional costs (Crosmary et al., 2012; Dooley & Judge, 2015).

Not surprisingly, top predator resting behavior was also influenced by humans and, to a lesser extent, bottom‐up factors. The influence of humans on top predators is evident from many species becoming more nocturnal in an increasingly urbanized world (Gaynor et al., 2018; Moll et al., 2018). In contrast, mesopredators face the challenge of balancing threats from top predators as well as acquiring resources from mobile prey. In some cases, both their predators and prey are active during overlapping hours, meaning they must choose between high‐risk, high‐reward foraging and low‐risk, low‐reward resting (Dias et al., 2019). For example, weasels *Mustela altaica* fine‐tune temporal dynamics to forage for pikas while avoiding foxes (Bischof et al., 2014).

These changes in predator resting patterns can have cascading impacts down the food chain. For example, if predator species shift their inactivity cycles to avoid humans, the original predator–prey synchrony patterns can dissolve (Martin‐Diaz et al., 2018). This phenomenon has been observed most in locations near human habitation and presence. For example, intensive hunting of moose *Alces alces* populations causes asynchronous patterns as the moose react more strongly to humans than their natural predators, wolves *Canis lupus*, which are relatively less abundant (Eriksen et al., 2011). With growing nocturnality in top predators (Gaynor et al., 2018), herbivores are caught in a constant temporal threat from diurnal human activity and nocturnal predation. For example, primarily diurnal mountain gazelle *Gazella gazella* that previously sought haven from predation in the daytime are now trapped by a diurnal predation threat from both increasing human presence and urbanization and nocturnal predation from their main predator, the golden jackal *Canis aureus* (Shamoon et al., 2018). Similarly, roe deer *Capreolus capreolus* in Europe, a primarily crepuscular species, are also now responding to indirect human cues and becoming more nocturnal. However, this directly overlaps with the activity pattern of their main nocturnal predator, the Eurasian lynx *Lynx lynx*, and the deer now face a constant temporal threat (Bonnot et al., 2020; Martin‐Diaz et al., 2018). Although previous studies suggest that highly vigilant prey species can maintain normal food intake levels for short durations, long‐term vigilance can negatively impact fitness (Fardell et al., 2020; Fortin et al., 2004). Because we found that temporal predator avoidance is the primary resting pattern across all mammalian orders, this diel predation threat may be a cause for concern.

### 4.2 Trophic levels and drivers

We found that lower trophic levels and smaller species such as rodents and lagomorphs frequently used spatial and social resting strategies, whereas top predators and larger species frequently used temporal resting strategies. Differences across trophic levels likely reflect a difference in the mobility of their food resources as well as the importance of predation as a cause of mortality. Herbivores' food resources are relatively constant across space and time, enabling these species to alter their behavior based on the activity patterns of their predators, including humans (Daly et al., 1992; Pratas‐Santiago et al., 2017) (Figure 5). For example, wild sloths *Bradypus variegatus* show a preference for resting at night, as their stable food resource allows them greater temporal flexibility to avoid predation, which supports the risk allocation hypothesis (Voirin et al., 2014). Similarly, small mammals such as the Indian crested porcupine *Hystrix indica* rest in spatial refugia during moonlit nights to reduce predation (Alkon & Saltz, 1988). Tamarins and lorisiforms also reduce predation risk by resting in nests during dawn or dusk (Franklin et al., 2007; Svensson et al., 2018).

## 5 CONCLUSION

While our study primarily focuses on intrinsic and extrinsic traits that relate to resting strategies, we did not consider the influences of long‐term processes such as genetic chronotypes, and sensory adaptations (Zielinski, 1988), as well as complex biological rhythms and community interactions (Lima et al., 2005). Our analyses were further limited by biases in the representation of taxa, with some orders, such as *Carnivora* and *Primates*, being studied frequently while others, such as *Chiroptera*, had very few studies pertaining to resting patterns in the wild, relative to their taxonomic diversity. Future research should seek to study resting patterns and predation avoidance strategies of these lesser understood species, as well as the interaction between short‐ and long‐term drivers of resting strategies.

## CONFLICT OF INTEREST

None declared.

## AUTHOR CONTRIBUTIONS

**Ishana Shukla:** Conceptualization (equal); data curation (lead); writing–original draft (lead); writing–review and editing (equal). **A. Marm Kilpatrick:** Formal analysis (equal); methodology (equal); visualization (equal); Writing–review and editing (equal). **Roxanne Beltran:** Conceptualization (equal); formal analysis (equal); methodology (supporting); supervision (lead); visualization (lead); writing–review and editing (equal).

## Supporting information

Supplementary Material

## Data Availability

Data are available in the Dryad repository at: https://doi.org/10.7291/D1XM3B.
