# Supplementary material for: Variation in resting strategies across trophic levels and habitats in mammals
Source: Ecol Evol. 2021 Oct 6;11(21):14405–15. doi: 10.1002/ece3.8073 (PMC8571619; doi:10.1002/ece3.8073)
Supplement: Supplementary file 1 — Supplementary Material [file ECE3-11-14405-s001.docx]

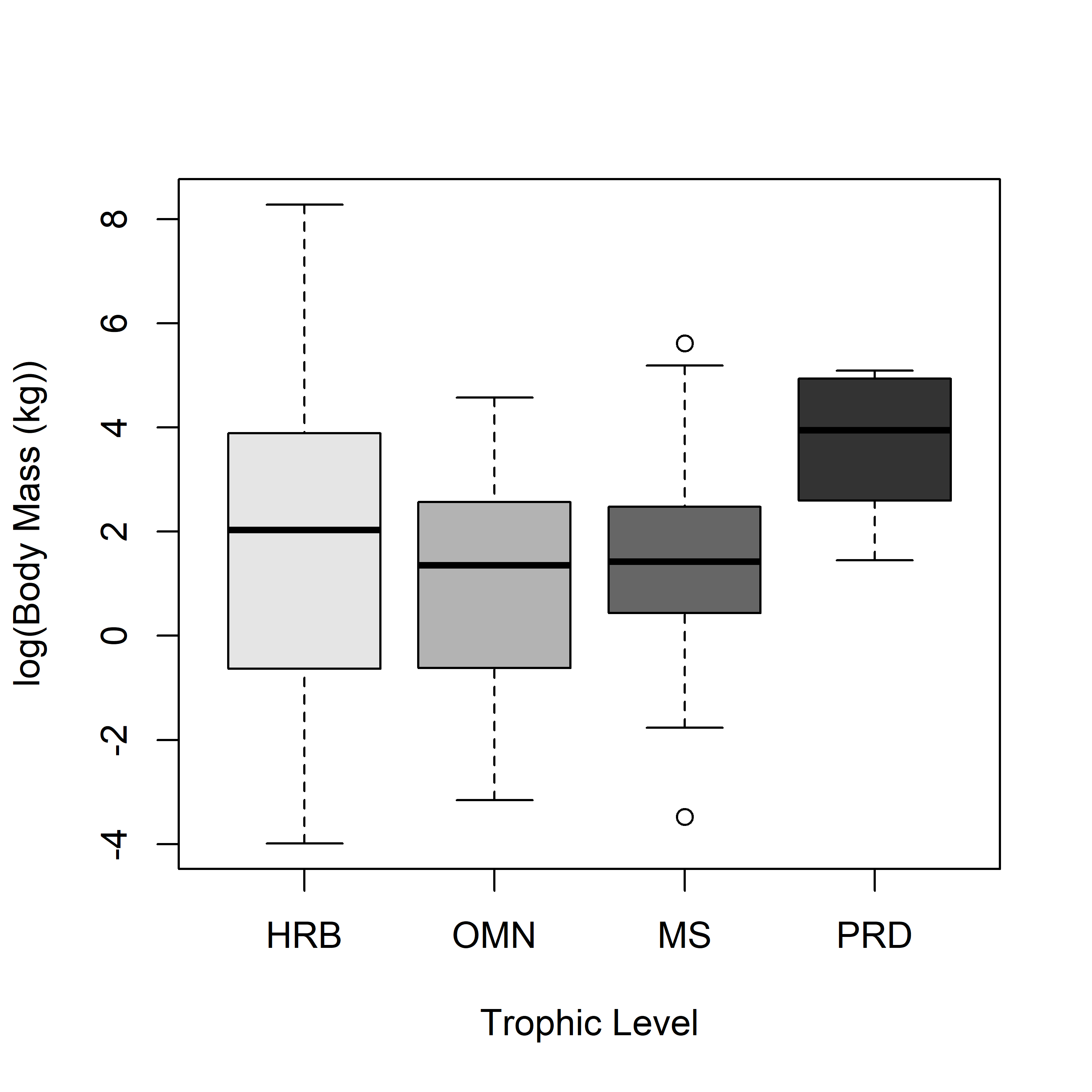


**Figure S1.** Body mass of top predators (PRD) was significantly larger than mesopredators (MS), omnivores (OMN), and herbivores (HRB) before accounting for phylogenetic relatedness.

**Table S1. Coefficients, standard errors, and 95% confidence intervals for resting strategy with mass (log-transformed) as a predictor from a Bayesian multinomial model with a phylogenetic variance-covariance matrix. Averages from ten phylogenetic trees are shown (the coefficient of variation of coefficient estimates among trees averaged 8.6%). The multinomial reference level is Social strategy.**

| Coefficient | Estimate | SE | Lower 95% CI | Upper 95% CI |
| --- | --- | --- | --- | --- |
| Spatial strategy Intercept | 1.15 | 0.94 | -0.71 | 3.15 |
| Spatial strategy Mass Slope | -0.28 | 0.13 | -0.54 | -0.03 |
| Temporal strategy Intercept | 0.54 | 2.10 | -3.83 | 4.57 |
| Temporal strategy Mass Slope | -0.10 | 0.16 | -0.42 | 0.21 |

**Table S2. Coefficients, standard errors, and 95% confidence intervals for resting strategy with trophic level as a predictor from a Bayesian multinomial model with a phylogenetic variance-covariance matrix. Averages from ten phylogenetic trees are shown (the coefficient of variation of coefficient estimates among trees averaged 11.6%). The multinomial reference level is Social strategy.**

| Coefficient | Estimate | SE | Lower 95% CI | Upper 95% CI |
| --- | --- | --- | --- | --- |
| Spatial strategy Intercept | 0.56 | 1.10 | -1.47 | 2.97 |
| Spatial strategy Mesopredator | 0.25 | 0.97 | -1.74 | 2.06 |
| Spatial strategy Omnivore | 0.60 | 0.68 | -0.78 | 1.89 |
| Spatial strategy Top predator | -7.92 | 16.01 | -40.76 | 21.70 |
| Temporal strategy Intercept | 0.68 | 1.65 | -2.93 | 3.84 |
| Temporal strategy Mesopredator | 0.46 | 1.12 | -1.67 | 2.82 |
| Temporal strategy Omnivore | -0.73 | 0.89 | -2.46 | 1.09 |
| Temporal strategy Top predator | 19.36 | 12.11 | 2.57 | 47.40 |

**Table S3. Coefficients, standard errors, and 95% confidence intervals for resting strategy with habitat as a predictor from a Bayesian multinomial model with a phylogenetic variance-covariance matrix. Averages from ten phylogenetic trees are shown (the coefficient of variation of coefficient estimates among trees averaged 14.6%). The multinomial reference level is Social strategy.**

| Coefficient | Estimate | SE | Lower 95% CI | Upper 95% CI |
| --- | --- | --- | --- | --- |
| Spatial strategy Aquatic | -0.51 | 1.44 | -3.24 | 2.46 |
| Spatial strategy Desert | -1.28 | 1.20 | -3.78 | 1.00 |
| Spatial strategy Grassland | 0.94 | 1.56 | -1.73 | 4.44 |
| Spatial strategy Savannah | -1.62 | 0.86 | -3.35 | 0.05 |
| Spatial strategy Temperate forest | 0.07 | 0.94 | -1.68 | 1.98 |
| Spatial strategy Urban | 10.05 | 8.53 | -1.95 | 29.84 |
| Spatial strategy Intercept | 1.31 | 1.18 | -0.87 | 4.03 |
| Temporal strategy Aquatic | -4.47 | 2.55 | -10.09 | 0.02 |
| Temporal strategy Desert | -0.91 | 1.44 | -3.87 | 1.84 |
| Temporal strategy Grassland | 1.74 | 1.76 | -1.43 | 5.63 |
| Temporal strategy Savannah | -1.55 | 1.16 | -3.99 | 0.60 |
| Temporal strategy Temperate forest | -0.45 | 1.29 | -3.10 | 1.98 |
| Temporal strategy Urban | 11.55 | 8.52 | -0.54 | 31.37 |
| Temporal strategy Intercept | 0.56 | 2.41 | -4.46 | 5.45 |
